# Functionalizing Collagen with Vessel‐Penetrating Two‐Photon Phosphorescence Probes: A New In Vivo Strategy to Map Oxygen Concentration in Tumor Microenvironment and Tissue Ischemia

**DOI:** 10.1002/advs.202102788

**Published:** 2021-08-19

**Authors:** Cheng‐Ham Wu, Kristina S. Kisel, Muthu Kumar Thangavel, Yi‐Ting Chen, Kai‐Hsin Chang, Ming‐Rung Tsai, Chia‐Yu Chu, Yu‐Fang Shen, Pei‐Chun Wu, Zhiming Zhang, Tzu‐Ming Liu, Janne Jänis, Elena V. Grachova, Julia R. Shakirova, Sergey P. Tunik, Igor O. Koshevoy, Pi‐Tai Chou

**Affiliations:** ^1^ Department of Chemistry National Taiwan University Taipei 10617 Taiwan; ^2^ Department of Chemistry University of Eastern Finland Joensuu 80101 Finland; ^3^ St.‐Petersburg State University 7/9 Universitetskaya nab St.‐Petersburg 199034 Russia; ^4^ Department of Dermatology National Taiwan University Hospital and National Taiwan University College of Medicine Taipei 10002 Taiwan; ^5^ Department of Bioinformatics and Medical Engineering Asia University Taichung City 41354 Taiwan; ^6^ 3D Printing Medical Research Institute Asia University Taichung City 41354 Taiwan; ^7^ Institute of Translational Medicine, Faculty of Health Sciences Ministry of Education Frontiers Science Center for Precision Oncology University of Macau Taipa Macau 999078 China

**Keywords:** phosphorescence lifetime imaging microscopy, phosphorescent oxygen sensors, Re^I^ diimine carbonyl complexes, tissue ischemia, tumor hypoxia, two‐photon phosphorescence

## Abstract

The encapsulation and/or surface modification can stabilize and protect the phosphorescence bio‐probes but impede their intravenous delivery across biological barriers. Here, a new class of biocompatible rhenium (Re^I^) diimine carbonyl complexes is developed, which can efficaciously permeate normal vessel walls and then functionalize the extravascular collagen matrixes as in situ oxygen sensor. Without protective agents, Re^I^‐diimine complex already exhibits excellent emission yield (34%, *λ*_em_  = 583 nm) and large two‐photon absorption cross‐sections (*σ*_2_  = 300 GM @ 800 nm) in water (pH 7.4). After extravasation, remarkably, the collagen‐bound probes further enhanced their excitation efficiency by increasing the deoxygenated lifetime from 4.0 to 7.5 µs, paving a way to visualize tumor hypoxia and tissue ischemia in vivo. The post‐extravasation functionalization of extracellular matrixes demonstrates a new methodology for biomaterial‐empowered phosphorescence sensing and imaging.

## Graphical abstract

Biocompatible rhenium (Re^I^) diimine carbonyl complexes functionalize the extravascular collagen matrixes as in situ oxygen sensor. After tail‐vein delivery and vessel penetration, the probe can bind extravascular collagens and achieve stabilization enhanced two‐photon phosphorescence yields. Increased deoxygenated phosphorescence lifetime from 4.0 to 7.5 µs paves a way to visualize tumor hypoxia and tissue ischemia in vivo.

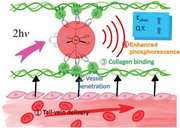

## 1 Introduction

Sufficient oxygen (O_2_) supply is critical to maintaining an organism's homeostasis. Chronically hypoxic niche of poorly perfused tissues may transform tumor biology to develop drug resistance, promote cancer cell stemness, invasion, metastasis, dormany, and enhance the clonal heterogeneity.^[^ ^1^ ^]^ Satellite tumors around in this transitional microenvironment may be neglected in the surgical removal of major sites. Measuring the O_2_ concentration in these biomedical contexts could help to understand the dynamics of pathophysiology in situ and identify the problematic niches for the relapse of cancers. Conventionally, the measurement of tissue oxygen levels relies on O_2_ microelectrodes.^[^ ^2^ ^]^ Despite the accuracy and immediacy, invasive insertion of an electrode stimulates the studied organ and causes inevitably metabolic disorders (i.e., bleeding induced micro‐inflammations). That artifact may change the physiological status and the actual read‐out of the O_2_ level. Besides, the lack of spatial information limits the use to navigate O_2_ distribution in vivo.

To achieve the least invasive mapping of O_2_ concentration in biomedical microenvironments, chemists have developed many transition metal complexes.^[^ ^3^, ^4^, ^5^, ^6^, ^7^, ^8^, ^9^, ^10^, ^11^, ^12^, ^13^ ^]^ Their long T_1_→S_0_ phosphorescence lifetime allows the collisional quenching by dissolved oxygen molecules. The quenching rate is proportional to O_2_ concentration, and the corresponding partial oxygen pressure (pO_2_) could be optically read‐out by phosphorescence lifetime measurement. Combined with laser‐scanned microscopes, the phosphorescence lifetime imaging microscopy (PLIM) can further map the microscopic distribution of oxygen in vivo.^[^ ^14^, ^15^, ^16^, ^17^, ^18^, ^19^, ^20^, ^21^, ^22^, ^23^ ^]^ For increased measurement depth, least photodamage, and high spatial resolution, near‐infrared (NIR) excited two‐photon (TP) and three‐photon phosphorescence probes were developed for in vivo oxygen sensing.^[^ ^9^, ^18^, ^19^, ^24^, ^25^, ^26^ ^]^ Strongly emissive complexes of Pt^II^,^[^ ^14^, ^27^, ^28^, ^29^ ^]^ Ru^II^,^[^ ^30^ ^]^ Eu^III^,^[^ ^31^, ^32^, ^33^ ^]^ and heterometallic Ir–Eu^[^ ^34^ ^]^ dyads commonly show rather poor (≈100 GM) two‐photon absorption (TPA) cross‐sections (*σ*2). The TPA could be significantly enlarged by coupling the metal ions with extended two‐photon absorbing organic ligands in a metal‐perturbed (D–*π*)n–A architectures.^[^ ^35^, ^36^, ^37^, ^38^, ^39^, ^40^ ^]^ Recently, large TPA and high quantum yields (Q.Y.) are simultaneously achieved in metalloporphyrin complexes.^[^ ^41^, ^42^ ^]^

However, probes' phosphorescence properties could be easily affected by molecules' structural rigidity, solvent viscosity, environment temperatures, and pH values of solutions. The binding of probes on surrounding proteins could easily change the rate of oxygen quenching. Besides, some of the probes are hydrophobic and not biocompatible if directly applied. Under these considerations, the biomedical phosphorescence probes usually need an encapsulation or surface modification to stabilize the performance and reduce the toxicity. This protection layer unavoidably enlarges the hydrodynamic sizes up to 5–100 nm,^[^ ^43^ ^]^ which is >5‐nm intercellular cleft of non‐fenestrated capillary and 1‐nm tight junctions in the brain and spinal cord.^[^ ^44^, ^45^ ^]^ As a result, many of them need to be injected at or near the studied site with minimally invasive surgery instead of systemically intravenous administration. Least‐invasive delivery can only be achieved either through ligand installation, enhanced vessel permeation in the tumor microenvironment or fenestrated capillaries of bone marrow cavities.^[^ ^19^ ^]^ This encapsulation or surface modification reduces the trans‐vascular permeability and restricts their broad applications in biomedicine.

To overcome this last‐mile hurdle of non‐invasive probe delivery, in this work, we design a new class of hydrophilic Re^I^‐diimine luminescent complexes employing water‐soluble phosphine ligands and collagen‐targeting sulfonic groups. Instead of pre‐encapsulation or dendrimer surface modification, we plan to let the non‐toxic probes penetrate the vessels first and then stabilized with extracellular matrixes. Despite a simple configuration of the target compounds, accessible modulation of their coordination sphere allowed to simultaneously achieve large two‐photon absorption (*σ*2 = 300 GM) and intense triplet luminescence (Φem = 0.34) in deoxygenated water at room temperature. The deoxygenated phosphorescence lifetime at the 37°C body temperature is insensitive to the pH value. It keeps around 4 µs in water, which is long enough to sense the change of pO_2_ in the microenvironment. The in vitro cell assay and in vivo animal studies showed good biocompatibility and dispersed circulation in angiography. Most importantly, without the protective carriers and encapsulation, the Re^I^‐diimine luminescent probes can easily penetrate typical vessel walls and diffuse into the dermis space of tissues. Owning to the high‐affinity of sulfonic groups to collagens, a mechanism typically used in histopathological labeling,^[^ ^46^ ^]^ the probe is stabilized on tissue collagens after extravasation. Considerable enhancement in Q.Y. and a prolonged lifetime (7.5 µs) in deoxygenated conditions are achieved. No other TP phosphorescence probes ever achieved such kind of biomaterial enabled stabilization after extravasation. With high two‐photon phosphorescence yields, properly long phosphorescence lifetime, high Stern–Volmer quenching constant for oxygen, good biocompatibility, and high vessel permeability, we believe the holistically advanced Re^I^‐diimine luminescent probes have great advantages to be applied in the in vivo studies of cell metabolism,^[^ ^16^, ^20^, ^21^ ^]^ stem cell niche,^[^ ^19^ ^]^ tumor hypoxic niche in poorly‐perfused tissues,^[^ ^22^ ^]^ blood perfusion,^[^ ^17^, ^18^ ^]^ or oxygen transportation.^[^ ^23^ ^]^

## 2 Results and Discussion

### 2.1 Synthesis and Characterization

Among the family of Re^I^ diimine carbonyl complexes, those with phenanthroline ligand are capable of showing higher phosphorescence quantum yields in comparison to their bipyridine congeners.^[^ ^47^, ^48^ ^]^ In particular, the dicarbonyl derivatives of [Re(phen)(CO)_2_(PR_3_)_2_]^+^ reach the values of Φ_em_ above 0.4 in fluid medium at room temperature.^[^ ^49^ ^]^ The photophysical properties of these species in solution were shown to be poorly sensitive to the electronic and stereochemical features of structurally similar ancillary phosphine ligands. Thus, in order to introduce hydrophilic groups with minimum interference into the optical characteristics of the {Re(phen)} chromophore, we have chosen two water‐soluble phosphines, 1,3,5‐triaza‐7‐phosphaadamantane (PTA) and Na_3_‐tris‐(3‐sulfophenyl)phosphine (TPPTS), which have been earlier successfully applied for the aqua‐solubilization of the photoactive organometallic compounds.^[^ ^50^, ^51^, ^52^ ^]^

**Scheme 1:**
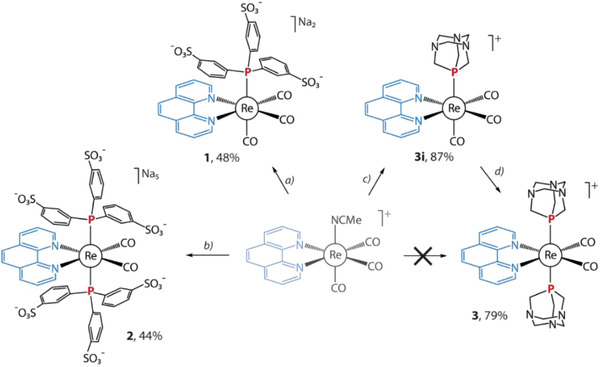
Synthesis of complexes 1–3 (*a*: TPPTS, water/ethanol 1:1 v/v, autoclave, 130 °C, 12 h, N_2_; *b*: TPPTS, water/ethanol 1:1 v/v, autoclave, 220 °C, 12 h, N_2_a; *c*: PTA, toluene, reflux, 12 h, N_2_; *d*: PTA, ONMe_3_^.^2H_2_O, acetone, reflux, 12 h, N_2_.

The TPPTS relatives, [Re(phen)(CO)_3_(TPPTS)]Na_2_ (**1**) and [Re(phen)(CO)_3_(TPPTS)_2_]Na_5_ (**2**) were prepared in an autoclave in water/ethanol mixtures at 130 and 220 °C, respectively and were isolated as polyanionic sodium salts (**Scheme** **1**). The mass spectra of **1** and **2** were recorded in a negative mode and revealed the peaks at *m/z* 972.87 (**1**) and 1512.75 (**2**), which correspond to the monoanionic [Re(phen)(CO)_3_(TPPTS)Na]^−^ and [Re(phen)(CO)_2_(TPPTS)_2_Na_4_]^−^ (Figure S1). The NMR data obtained for **1** and **2** are completely compatible with the structural patterns suggested in Scheme 1. Each of the ^31^P NMR spectra displays a single resonance indicating that mono‐(**1**) and di‐ substituted phosphine compounds (**2**) correspond to C_s_ (**1**) and C_2v_ (**2**) symmetry point groups. The ^1^H NMR spectroscopic patterns, see the Experimental section, also fit well these structural hypotheses.

The above synthetic approach works well for the anion type of Re^I^ complexes. For reference, we also synthesized the cationic type compound **3** (see Scheme 1), and its monophosphine predecessor **3i**, which has been described previously.^[^ ^52^, ^53^ ^]^ Both ^1^H and ^31^P NMR data fit well the composition and structure of **3**.

### 2.2 Photophysical Properties

**Figure 1:**
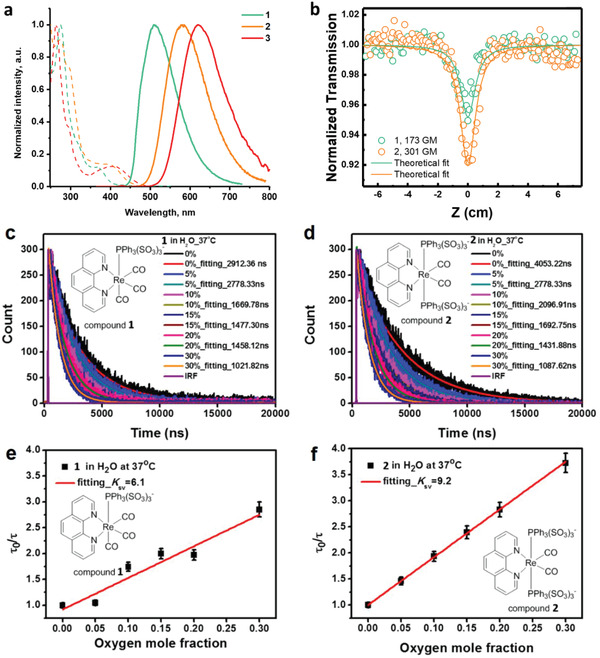
a) Normalized absorption (dashed line) and emission (solid line) spectra of 1–3 in water. *λ*_ex_ = 375 nm, b) The z‐scan experimental data of 1 (green circles) and 2 (orange circles) in neutral water. Samples were held in a cuvette cell with 1‐mm thickness. Solid red lines are the corresponding fit of the data points (pulse energy = 1.80 µJ, pulse width: 180 fs). *λ*_ex_ = 800 nm. c–f) Dependence of phosphorescence lifetime on oxygen concentration for complex 1 and 2 (PBS buffer, 37 °C), respectively. The *τ*_0_ represents the phosphorescence lifetime at 0% oxygen concentration, *K*_SV_ – Stern‐Volmer quenching constants. The phosphorescence of 1 and 2 were single‐photon excited at 375 nm and detected at around 500 nm.

The optical behavior of the title complexes was studied at room temperature in water solutions and in the solid‐state; the pertinent data are summarized in **Table** **1**. Absorption spectra of **1** and **2** (**Figure** **1**a) display intense bands in the region 260–280 nm, which can be assigned to the *π*–π* transitions localized at the aromatic system of the phen ligand. The lower energy broad absorptions around 365–404 nm are assigned to Re→phen charge transfer (^1^MLCT), which shows a bathochromic shift upon the increase of the number of Re‐bound phosphines. This redshift is further enhanced by the aliphatic PTA versus a triaryl TPPTS, a similar effect was noticed for congener compounds with PEt_3‐_ *_x_* Ph*_x_* ligands.^[^ ^54^ ^]^

**Table 1:** Photophysical properties of 1–3 in H_2_O (pH = 7.4) and in solid‐state at 310 K

H_2_O Solution						Solid		
	*λ*_abs_ [nm] [*ε*×10^–3^, M^–1^cm^–1^]	*λ*_em_ [nm]	*Φ*_em_ ^a)^	*Φ*_em_ ^b)^	*τ* ^b)^, µs	*λ*_em_ [nm]	*τ*, µs	*Φ*_em_
1	366 (2.6); 319 (6), 275 (22)	515	0.05	0.10	2.91	540	1.21	0.03
2	379 (3.8); 296 (20); 271 (25)	583	0.15	0.34	4.05	537	9.30	0.30
3	404 (2.6); 293 (8); 263 (22); 221 (28)	634	0.02	0.03	0.33	645	2.19	0.04

^a)^ Aerated;

^b)^ degassed solution;

^c)^ excitation wavelengths *λ*_ex_ = 375 nm.

The emission energies of the studied complexes in solution are in line with those observed in absorption spectra and with the trends typical for the Re diimine luminophores, where an increase in the number of phosphine ligands in the coordination sphere or in their donor ability (alifatic versus aromatic phosphines) leads to a significant red shift of emission maxima.^[^ ^54^ ^]^ Expectedly, the dicarbonyl compounds **2** and **3** exhibit visibly red‐shifted luminescence than their tricarbonyl monophosphine relatives **1** and **3i** in both solution and solid phase.^[^ ^49^, ^55^, ^56^ ^]^ The luminescence intensity for these species in solid is approximately as strong as that in the degassed solution. The microcrystalline complex **2** shows high quantum efficiency (Φ_em_ = 0.3), nearly an order of magnitude larger than that of **1**. In solution, the lifetimes for **1** and **2** span the range from 2.91 to 4.14 µs (Figure 1c–f) at room temperature that points to a triplet character of the observed emission, assigned predominantly to the metal‐to‐ligand charge transfer (^3^MLCT [*d_*π*_* (Re)→ππ*(phen)]. Upon aeration, the quantum yield of **2** in water solution drops from 0.34 to 0.15, accompanied by a visible shortening of the lifetime (from 4.05 to 1.43 µs, Figure 1d,f). This phosphorescence property indicates collisional quenching by molecular oxygen via the triplet‐triplet annihilation pathway.

As for the reference compound **3**, the emission peaks are at 634 nm in solution and 645 nm in the solid phase. Because **3** turns out to be non‐selectively spread in tissue fluid (vide infra), it is used as a reference compound to support the mechanism of bioconjugation suggested for **1** and **2**. Therefore the photophysical and nonlinear properties of **3** are not elaborated in detail.

### 2.3 Biomedical Oxygen Sensing and Imaging

High photostability of **2**, intense triplet emission in water, and excellent water solubility prompted us to investigate its bio‐application in the mapping of oxygen concentration in vitro and in vivo. First of all, we established the relationship between the phosphorescence lifetime and O_2_ concentration by the Stern–Volmer equation:

(1) $$\frac{\tau_{0}}{\tau }=1+K_{\mathrm{sv}}\left[O_{2}\right]$$

where *τ*_0_ represents the phosphorescence lifetime under O_2_‐free conditions, and *K*_SV_ is the quenching constant. In the physiological medium (PBS buffer, pH = 7.4 at 37 °C), the Stern–Volmer plots of **1** and **2** are approximately linear in the range of 0–30% oxygen mole fraction in 1 atm gas, and the *K*_SV_ values are 6.1 and 9.2 atm^–1^, respectively (Figure 1c–f), which indicate a moderate quenching sensitivity. We also investigated the temperature and pH dependence of phosphorescence lifetime (Figure S3, Supporting Information). The data obtained indicate that lifetime decreases as temperature increases. In contrast, the lifetime of **2** is insensitive to the pH variations in the range from 5.1 to 8.5. (Figure S3c, Supporting Information), that prevent the lifetime signal crosstalking and is evidently beneficial to the oxygen sensing.

For deep‐tissue in vivo bio‐imaging and sensing, the probe needs to have strong two‐photon absorption and efficient two‐photon phosphorescence. We investigated the two‐photon absorption properties of **1** and **2** in pure water by the open‐aperture Z‐scan method.^[^ ^57^ ^]^ Upon fitting the data (Figure 1b), the two‐photon absorption cross‐section 2 (was then calculated to be 173 ± 7 GM and 300 ± 5 GM for **1** and **2**, respectively. These values are comparable to that of Coumarin 480 of 148 GM in ethanol upon excitation at 800 nm.^[^ ^57^ ^]^

To ensure their biocompatibility, we carried out the MTT test to assess the viability of human liver carcinoma (HepG2) cells. Incubating cells with the probes for 15 hours, we observed no obvious cytotoxicity even at a high concentration up to 500 µm (Figure S4, Supporting Information). We also confirmed the uptake of probes by HepG2 cells via bright‐field, two‐photon phosphorescence images and the corresponding two‐photon luminescent spectra, which are shown in Figure S5, Supporting Information. The intracellular luminescence spectra fit well those obtained for **1** and **2** in an aqueous solution. These observations clearly indicate that probes **1** and **2** are biocompatible and could be successfully internalized by cells.

To verify the collagen‐targeting specificity, as a first step, we measured the phosphorescence lifetime of **2** in various biological media in vitro. Results showed that phosphate buffer saline (PBS), albumin solution and fetal bovine serum (FBS) did not change the lifetime properties a lot (Table S1, Supporting Information) either at 0% O_2_ or at 20% O_2_ condition. The pure Matrigel, containing extracellular matrix proteins, slightly decreases the lifetime at 0% O_2_, which may be due to the change of solution viscosity. We then mixed the Matrigel with lipopolysaccharide (LPS;10 ng mL^−1^), interferon‐Gama (IFN‐*γ*; 20 ng mL^−1^) and 30% L929 fibroblast conditioned medium and plugged it into the abdomen space of a C57BL/6‐C2J mouse. On the 7th day post‐implantation, we took the Matrigel out for imaging. As we previously demonstrated, the Matrigel recruited M1 macrophages and stimulated the growth of collagen fibers (Figure S6a, Supporting Information) inside it.^[^ ^58^ ^]^ Administrating 100 µm complex **2** into the thus prepared Matrigel, the region of collagen fibers (green color in Figure S6b, Supporting Information) exhibited strong two‐photon phosphorescence, indicating an enhanced quantum yield of **2** on the collagen fibers. Analyzed by two‐photon phosphorescence lifetime imaging microscopy (TP‐PLIM), we found that the collagen‐bounded lifetime was obviously prolonged to 6 µs at 20% O_2_ ambient condition and to 6.5 µs under nitrogen purged condition (Figure S6c,d, Supporting Information). These results demonstrated that complex **2** could be stabilized by the affinity binding to collagens and the phosphorescence yields and lifetimes were enhanced significantly.

**Figure 2:**
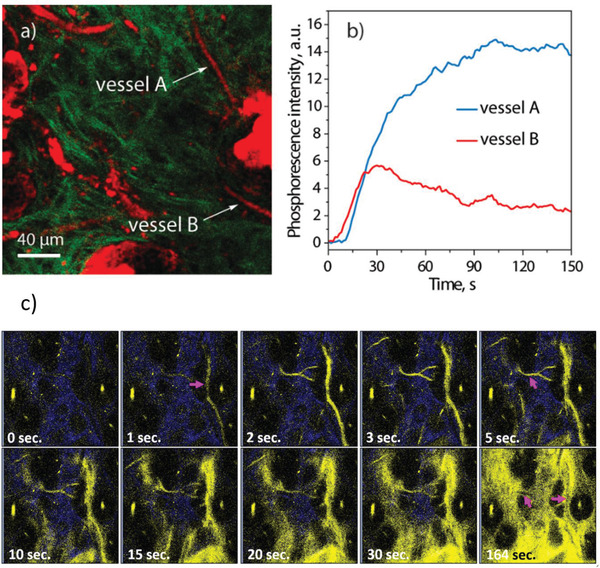
a) Second harmonic generation (green) and 550–650 nm two‐photon luminescence (red) images in mice skin under the excitation at 800 nm. White arrows indicate two different blood vessels A and B appeared after injection of complex **2**. b) Kinetics of the two‐photon phosphorescence intensities in the blood vessel A and B after injecting the complex **2**. c) The flush‐in and extravasation of complex **2** in vivo recorded by 800 nm excited two‐photon luminescence imaging. Blue color represents the SHG signals from collagens. (Blood vessel: magenta arrow). Fields of view: 240 × 240 µm.

To investigate the delivery kinetics and the TP‐PLIM performance of Re^I^‐complex in vivo, we first administered the brighter complex **2** into c2J mice through the tail vein injection. According to the experimental protocol,^[^ ^17^ ^]^ we mounted the mice's ear on the microscope stage and located the vessels before injection. After the intravenous administration, the 800‐nm excited two‐photon phosphorescence of the complex **2** in vessels can be clearly observed (**Figure** **2**a), and their flush‐in kinetics were time‐course recorded as well (Figure 2b).

**Figure 3:**
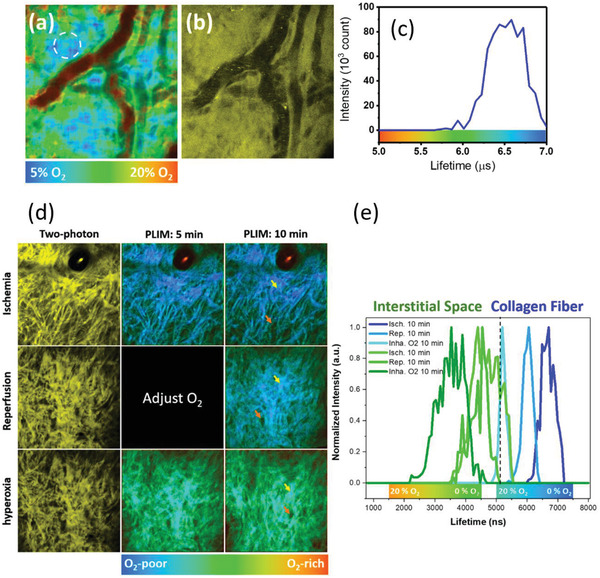
The in vivo two‐photon a) PLIM and b) luminescence intensity image (yellow) of the Re^I^‐complex **2** in mice ear after the tail‐vein injection. Fields of view: 100 × 100 µm. c) The corresponding histogram of phosphorescence lifetime in dense collagen networks [dashed circle in (a)] were typically around 6.5 µs. d) In vivo two‐photon phosphorescence (yellow) and PLIM of Re^I^‐complex **2** at 5 and 10 min after ischemia, reperfusion of ischemia, and hyperoxia; Fields of view: 240 × 240 µm. e) The lifetime histograms of PLIM at the collagen region (yellow arrow) and interstitial space (orange arrow) at different oxygenation conditions (Isch – ischemia, Rep – reperfusion, Inha – inhalation).

It is known that under a normal physiological condition, the low oxygen level blood in veins returns to the heart, exchanges gas (i.e., oxygen and carbon dioxide) in the lung, and finally, the oxygenated blood is pumped by the heart for the next peripheral circulation. The blood vessel types can be determined by the phase of perfusion in angiography.^[^ ^59^ ^]^ Obviously, the phosphorescence signals appeared first in arterial vessel B with a signal level lower than those in venous vessel A due to the oxygen quenching. Owing to the small sizes of the phosphorescent molecules, the Re^I^‐complex **2** can quickly penetrate the vessel walls and distribute well in tissues within a time period as short as 3 min (Figure 2c). Due to the directional design, **2** tends to accumulate on the collagen networks that has been unambiguously revealed by a combination of SHG images and those obtained by using two‐photon excited emission of the probe (Figure S7, Supporting Information). It was found that under normoxia condition, in the regions close to the vessel, the in vivo phosphorescence lifetime image and corresponding histogram shows a peak at around 6.5 µs (**Figure** **3**a–c).

Similar to the in vitro results (Figure S7, Supporting Information), the lifetimes at collagens (yellow arrows in Figure 3d) tend to be longer than those in interstitial space (orange arrows in Figure 3d). The lifetime histograms of collagen‐associated probes are also narrower than those in interstitial areas, indicating a smaller inhomogeneous broadening of the lifetime signal (Figure 3e). These results indicate that **2** can be stabilized by the affinity binding to collagens, thus mitigating bio‐environment effects and enhancing its phosphorescence properties. Its retention time in collagen tissues could last for at least 6 h (Figure S8, Supporting Information). We then calibrated the phosphorescence lifetime at different oxygen level by administrating complex **2** to the collagen tissues ex vivo (Figure S9, Supporting Information). The tissue was excised from mice and bathed in normal saline at 33 °C. The oxygen level was adjusted by O_2_‐N_2_ gassing system and monitored by an O_2_ meter (500U, Unisence) during the calibration experiment. The histogram peak of the phosphorescence lifetime was found at 7.5 µs at 0% O_2_ and dropped to 5 µs at 21.5% O_2_. Following the *ex vivo* calibration curve, the 6.5 µs lifetime around the vessel corresponds to a 6.46% O_2_ (pO_2_ = 49.1 mm Hg) under tissue normoxia conditions. It is well‐known that the tissue oxygenation level highly depends on the blood flow and cellular consumption rate to give O_2_ concentration in normal oxygenation from 1% to 9.5%.^[^ ^60^ ^]^ Thus, the in vivo obtained lifetime value (6.5 µs) corresponds to oxygen concentration, which falls in the range typical for normoxia conditions.

**Figure 4:**
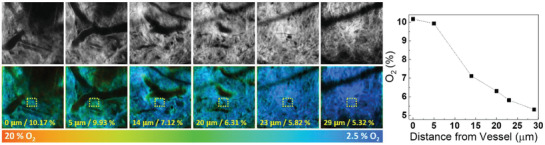
Two‐photon PLIM tomography of normal skin at various depths. The emission intensity of the complex **2** in tissue was shown in grayscale images. The vertical distance (in µm) from the giant vessel was recorded by the objective stage, and the corresponding oxygen concentration (estimated from the peak value in the lifetime histogram) was presented. Oxygen decreases rapidly as the distance increase. Fields of view: 240 × 240 µm.

To evaluate whether the probes can detect the variation of tissue oxygen levels in vivo, we induced partial hypoxia in the tissues by pressing the vessels at the root of the mice ear. Under these conditions, the tissues consume more oxygen than supply, and the phosphorescence lifetime histogram in PLIM finally showed an increased peak around 7 µs (Figure 3e). This result corresponds to a 22.8 mm Hg pO_2_ (3% O_2_). Since the circulation might not be fully blocked, the complete hypoxic conditions (i.e., 0% O_2_) was not reached. The oxygen pressure returned to a normal level of 53.2 mm Hg (PLIM histogram peaks at 6 µs) 10 min after reperfusion (Figure 3e). Accordingly, long‐term inhalation of ≈100% oxygen at 760 mm Hg gave peak of lifetime histogram downshifted to 5.25 µs (pO_2_ = 137 mm Hg; 18% O_2_). We then investigated the gradient of oxygen around the vessels. The oxygen concentration near large vessels (green color in **Figure** **4**) was 10% (pO_2_ = 76 mm Hg), which is obviously higher than those in the regions far away from the vessel (blue color in Figure 4).^[^ ^61^ ^]^ At a depth of 29 µm away from the vessel, the average oxygen concentration can be decreased down to 5% (pO_2_ = 76 mm Hg) level (Figure 4, right)^.[^ ^62^ ^]^

**Figure 5:**
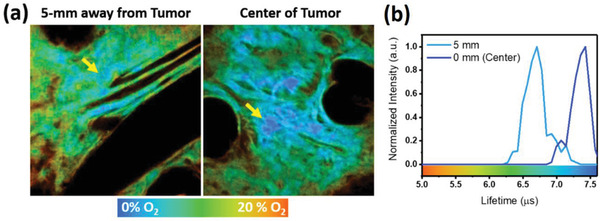
a) TP‐PLIM results of complex **2** at the center of tumor and 5‐mm away from tumor in a mouse's ear. b) Corresponding lifetime histograms measured around the region indicated by yellow arrow. Fields of view: 100 × 100 µm.

Finally, the hypoxia conditions in the melanoma microenvironment were carefully investigated (**Figure** **5**). We implanted mCherry labeled melanoma cells into the C57BL/6‐c2J (c2J) mice ear, and the tumor nodule appeared after 2–4 weeks. After locating the tumor position in vivo through the mCherry fluorescence, we tail‐vein injected the probes for the PLIM. We found pathological hypoxia (pO_2_ = 7.6 mm Hg; 1% O_2_) at the center of the tumor, where PLIM display the average lifetime of 7.32 µs. At the edge of the tumor, the oxygen tension increased to 22.8 mm Hg (7 µs; 3% O_2_) and up to 38.1 mm Hg at the region 5‐mm away from the edge (6.7 µs; 5% O_2_). These results agree well with the change in oxygen tension during tumor development.^[^ ^63^ ^]^

**Scheme 2:**
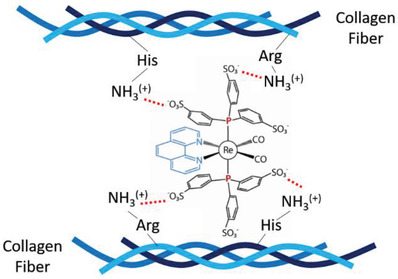
The proposed structure of **2** complex anchoring on collagen via electrostatic and hydrogen bonds. His: Histidine; Arg: Arginine.

Evidently, it is of particular importance to double‐check the reasons for the high binding affinity of **2** to collagens. For the sake of comparison, we performed angiography experiments with complexes **1**, **2,** and **3** and found they all penetrated the vessel walls. Two‐photon phosphorescence signals of **1** and **2** (Figure S10, Supporting Information, green and yellow colors, respectively) are co‐localized with the second harmonic generation signals of collagen fibers (blue colors in Figure S10, Supporting Information). In sharp contrast, complex **3** tends to spread evenly over the extravascular space without any clusterization. Because (**1**, **2**) and (**3**) contain the same 1,10‐phenanthroline and –CO ligands in their coordination sphere, the difference in collagen affinity cannot be attributed to these groups. A similar chemical design also excludes the intercalation of 1,10‐phenanthroline into the triple helix region of collagens. Therefore, most plausibly, the outer shell ionic properties account for this discrepancy. The most reasonable hypothesis might be that in the case of **1** and **2** the peripheral sulfonic anions act as tripods, which can anchor the complexes to collagen by either electrostatic interaction or hydrogen bonding with free amino or ammonium residues.^[^ ^64^ ^]^ This viewpoint can be envisaged by a cartoon‐like schematic diagram shown in **Scheme** **2**. On the other hand, the positive charge in the cationic complex **3** is located at the central Re ion, the peripheral ligand of which may only be the subject of a weak van der Waals interaction. As a result, **3** spreads evenly in the entire tissue fluid without specific affinity to collagen‐binding. Accordingly, we report an intriguing case that collagen can be functionalized by vessel‐penetrating probe **2** (and **1**), and then successfully applied in TP‐PLIM to visualize tumor hypoxia and tissue ischemia in vivo via oxygen mapping.

## 3 Conclusions

In this study, we developed a series of water‐soluble and biocompatible Re^I^‐diimine carbonyl complexes that can easily penetrate the vessel walls and detect oxygen tension in vitro and in vivo for a long period. Among these probes, complex **2** exhibits excellent two‐photon absorption cross‐sections (300 GM) under the excitation with an 800‐nm femtosecond laser. High two‐photon emission intensity allows visualizing the distribution of oxygen in the deep tissue regions. Moreover, a wide range of phosphorescence lifetime (∆*τ *≈ 3 µs) between oxygenated and deoxygenated conditions makes it sensitive enough to quantify the oxygenation in biological tissues, which is typically lower than 10%. Most importantly, complex **2** can easily permeate into tissues and form a stabilized bioconjugate with collagens for at least 6 h. This “**2**‐collagen” bioconjugate outperforms the starting complex with enhanced quantum yields and enlengthened phosphorescence lifetime. Since complex **2** was stuck on collagen and did not diffuse away quickly, it could serve as stationary oxygen sensors at local tissues. Then in the in vivo studies, we found that the phosphorescence lifetime of **2** increased from 5.25 µs nearby vessels to 7 µs in the avascular area. Based on the *ex vivo* calibration, the corresponding oxygen tension decreased from 10.17% to 5.32%, which agrees well with the oxygen level in skin tissue under physiological conditions.^[^ ^60^ ^]^ A lower level (<5%) of oxygen was found in pathological hypoxia. Lifetimes around 7.3 µs and 6.9 µs were found at the center and at the edge of the melanoma, corresponding to the oxygen level of 1% and 4%, respectively. These results suggest that the water‐soluble and biocompatible Re^I^‐complex **2** is, with the help of deep‐tissue TP‐PLIM, suitable for monitoring tissue metabolism, ischemia, and tumor hypoxia. This post‐extravasation stabilization and lifetime enlengthening by collagen render a new strategy for designing phosphorescence probes in biological tissues.

## 4 Experimental Section

### Synthesis General Comments

1,3,5‐Triaza‐7‐phosphaadamantane (PTA),^[^ ^65^ ^]^ [Re(phen)(CO)_3_(NCMe)](CF_3_SO_3_)^[^ ^66^ ^]^ were prepared according to published procedures. Toluene was distilled from Na‐benzophenone ketyl under nitrogen prior to use. Other reagents and solvents were used as received. The solution ^1^H and ^31^P{^1^H} spectra were recorded on a Bruker Avance 400 spectrometer. Mass spectra were measured on a Bruker APEX‐Qe Qh‐FT‐ICR instrument in ESI^–^ (**1**, **2**) modes. Microanalyses were carried out at the analytical laboratory of the University of Eastern Finland.

### [Re(phen)(CO)_3_(TPPTS)]Na_2_ (**1**)

[Re(phen)(CO)_3_(CH_3_CN)](CF_3_SO_3_) (200 mg, 0.312 mmol), TPPTS (176 mg, 0.31 mmol) and the mixture of water with ethanol (1:1 v/v, 15 mL) were placed in a 60 mL autoclave, pressurized with nitrogen to 25 atm and heated at 130 °C overnight. The resulting yellow solution was evaporated, the residue was dissolved in methanol and precipitated with acetone to give pale creamy flaky solid (160 mg, 48%). ESI‐MS (*m/z*): [M+Na]^−^ 972.87 (calcd 972.88). IR (CH_3_CN, *ν*(CO), cm^–1^). ^1^H NMR (methanol‐d_4_, 298 K; *δ*): 9.29 (d, *J*_HH_ 5.2 Hz, 2H, 2,9‐H phen), 8.67 (d, *J*_HH_ 8.2 Hz, 2H, 3,8‐H phen), 8.09 (s, 2H, 4,7‐H phen), 7.85 (dd, *J*_HH_ 5.2 and 8.2 Hz, 2H, 5,6‐H phen), 7.78 (d, *J*_HP_ 9.7 Hz, 6H, TPPTS), 7.31 (dd, *J*_HH_ 8.0 and 7.1 Hz, 3H, TPPTS), 7.13 (dd, *J*_HH_ 8.0 Hz, 3H, *J*_HP_ 9.4 Hz, TPPTS). ^31^P{^1^H} NMR (methanol‐d_4_, 298 K; *δ*): 21.2 (s). Anal. Calcd for C_33_H_20_N_2_Na_2_O_12_PReS_3_^.^H_2_O: C 39.08; H 2.62; N 2.76; S 9.70. Found: C 37.97; H 2.61; N 2.68; S 9.21.

### [Re(phen)(CO)_3_(TPPTS)_2_]Na_5_ (**2**)

[Re(phen)(CO)_3_(CH_3_CN)](CF_3_SO_3_) (118 mg, 0.184 mmol), TPPTS (262 mg, 0.461 mmol) and the mixture of water with ethanol (1:1 v/v, 15 mL) were placed in a 60 mL autoclave, pressurized with nitrogen to 25 atm and heated at 220 °C overnight. The resulting yellow solution was evaporated and the residue was washed with cold methanol (2 mL). Recrystallization by a gas phase diffusion of acetone into water solution of **2** at room temperature to afforded bright yellow fine crystalline material (125 mg, 44%). ESI‐MS (*m/z*): [M+Na_4_]^−^ 1512.75 (calcd 1512.85), [M–TPPTS+Na]^−^ 944.88 (calcd 944.94). IR (CH_3_CN, *ν*(CO), cm^–1^). ^1^H NMR (D_2_O, 298 K; *δ*): 8.43 (d, *J*_HH_ 5.0 Hz, 2H, 2,9‐H phen), 8.23 (d, *J*_HH_ 8.1 Hz, 2H, 3,8‐H phen), 7.83 (s, 2H, 4,7‐H phen), 7.60 (m, 6H, TPPTS), 7.41 (m, 6H, TPPTS), 7.30 (m, 12H, TPPTS), 7.17 (dd, *J*_HH_ 8.1 and 5.0 Hz, 2H, 5,6‐H phen). ^31^P{^1^H} NMR (D_2_O, 298 K; *δ*): 24.1 (s). Anal. Calcd for C_50_H_32_N_2_Na_5_O_20_P_2_ReS_6_^.^4H_2_O: C 37.33; H 2.50; N 1.74; S 11.96. Found: C 37.22; H 2.52; N 1.69; S 12.08.

### [Re(phen)(CO)_3_(PTA)](CF_3_SO_3_) (**3i**)

Re(phen)(CO)_3_(CH_3_CN)](CF_3_SO_3_) (500 mg, 0.78 mmol) and PTA (126 mg, 0. 803 mmol) were dissolved in toluene (60 mL). The mixture was refluxed overnight under a nitrogen atmosphere. The solvent was evaporated and yellow‐green solid was recrystallized by gas phase diffusion of diethyl ether into an acetone/methanol solution of **1** at room temperature to give yellow green crystalline material (510 mg, 87%). ESI‐MS (*m/z*): [M]^+^ 608.08 (calcd 608.08). IR (CH_3_CN, *ν*(CO), cm^–1^): 2041s, 1954s, 1928s. ^1^H NMR (acetone‐d_6_, 298 K; *δ*): 9.67 (dt, *J*_HH_ 5.2 and 1.2 Hz, 2H, 2,9‐H phen), 9.13 (ddd, *J*_HH_ 8.3, 1.2 and 0.9 Hz, 2H, 3,8‐H phen), 8.46 (s, 2H, 4,7‐H phen), 8.27 (dd, *J*_HH_ 5.2 and 8.3 Hz, 2H, 5,6‐H phen), 4.34 (d, *J*_HH_ 13.2 Hz, 3H, PTA), 4.25 (d, *J*_HH_ 13.2 Hz, 3H, PTA), 3.82 (s, 6H, PTA). ^31^P{^1^H} NMR (acetone‐d_6_, 298 K; *δ*): 82.0 (s). Anal. Calcd for C_22_H_20_F_3_N_5_O_6_PReS: C 34.92; H 2.66; N 9.26; S 4.24. Found: C 34.98; H 3.10; N 8.89; S 3.88.

### [Re(phen)(CO)_2_(PTA)_2_](CF_3_SO_3_)_2_ (**3**)

**3i** (100 mg, 0.132 mmol) and PTA (40 mg, 0. 255 mmol) were dissolved in acetone (15 mL), and the solution was degassed by three freeze‐pump‐thaw cycles. Then a solution of trimethylamine N‐oxide dihydrate (16 mg, 0.144 mmol) in an oxygen‐free acetone/methanol mixture (3 mL, 2:1 v/v) was added, turning the color from yellow to orange‐red. The reaction was refluxed overnight under a nitrogen atmosphere. Then the volatiles was evaporated, the residue was washed with toluene and diethyl ether. It was passed through a pad of alumina (neutral) in acetone/methanol/diethyl ether mixture (5:1:5 v/v/v), and recrystallized by a gas phase diffusion of diethyl ether into this solution (orange‐red block crystals, 93 mg, 79%). ESI‐MS (*m/z*): [M]^+^ 737.16 (calcd 737.16). IR (CH_3_CN, *ν*(CO), cm^–1^): 1944s, 1874s. ^1^H NMR (acetone‐d_6_, 298 K; *δ*): 9.68 (dm, *J*_HH_ 5.1 Hz, 2H, 2,9‐H phen), 9.01 (dm, *J*_HH_ 8.2 Hz, 2H, 3,8‐H phen), 8.39 (s, 2H, 4,7‐H phen), 8.16 (dd, *J*_HH_ 8.2 and 5.1 Hz, 2H, 5,6‐H phen), 4.33 (d, *J*_HH_ 12.5 Hz, 6H, PTA), 4.18 (d, *J*_HH_ 12.5 Hz, 6H, PTA), 3.63 (s, 12H, PTA). ^31^P{^1^H} NMR (D_2_O, 298 K; *δ*): 73.9 (s). Anal. Calcd for C_27_H_32_F_3_N_8_O_5_P_2_ReS: C 36.61; H 3.64; N 12.65; S 3.62. Found: C 36.67; H 3.69; N 12.71; S 3.59.

### Photophysical Measurements

UV‐vis absorption spectra were recorded with a Shimadzu UV‐1800 spectrophotometer; excitation and emission spectra in solution and in the solid‐state were recorded with a HORIBA FluoroMax‐4, lifetimes and emission quantum yields were measured on a HORIBA Scientific FluoroLog‐3 spectrofluorimeter. The absolute emission quantum yields in solution were determined using rhodamine 6G in ethanol (Φ_em_ = 0.95) as a standard. The uncertainty of the quantum yield measurement was in the range of ±5% (an average of three replicas). The emission decays were fitted by the sum of the exponential functions with a temporal resolution of 300 ps by deconvolution of the instrument response function. The measurement of the two‐photon absorption property has been elaborated in the previous report.^[^ ^66^ ^]^

### Bioanalytical Study

1) In vitro Cytotoxicity Assay: To assess the cytotoxicity of the complexes **1** and **2**, we treated them on the HepG2 cells and analyzed the cell viability by a colorimetric assay agent, 3‐(4,5‐dimethylthiazol‐2‐yl)‐2,5‐diphenyltetrazolium bromide (MTT, Roche). The HepG2 cells were seeded in a 96‐well plate with 5 × 10^3^ cells per well in a 90% Dulbecco's Modified Eagle Medium supplemented with 10% heat‐inactivated fetal bovine serum, penicillin, and streptomycin. To contrast with the control, six different concentrations of Re^I^‐complex were added to each well (highest to 500 µm). After 15 h of incubation, cells were washed twice with PBS buffer and then incubated with 200 µL of the culture medium with 10% MTT agent per well. After 4 h of reaction time, the culture medium was removed and refilled with 200 µL of dimethyl‐sulfoxide (Sigma‐Aldrich) per well to dissolve the purple MTT formazan crystal. The optical density of these samples was measured at 595 nm. All measurements were done with three replicates using an ELISA reader (VersaMax Microplate Spectrophotometers; Molecular‐Devices). The uptake of probes in HepG2 cells was also validated via two‐photon luminescence microscopy and spectroscopy under the illumination of an 800‐nm femtosecond laser.
2) Ex vivo Calibration: To calibrate the phosphorescence quenching rates of collagen‐bound **2**, we measured the TP‐PLIM of the complex **2** administered to the collagen tissue ex vivo (Figure S9, Supporting Information). The fresh collagen tissue was excised from a sacrificed mice's ear and then bathed in the normal saline with 100 µm complex **2** at 33 °C. The prepared sample in saline was held on a bottom‐glass petridish for 800 nm excited TP‐PLIM. The oxygen mole fraction in the measurement chamber was adjusted by O_2_‐N_2_ gassing system and monitored by an O_2_ meter (500U, Unisence) during the experiment. All the experiments were done within an hour. Finally, we generated the lifetime histogram of each PLIM image (Figure S9c, Supporting Information) and chose the peak lifetime as the representative one at different oxygen mole fraction (Figure S9b, Supporting Information).
3) Phosphorescence lifetime of probes in biological substances and Matrigel: In order to make sure it's the collagen that stabilizes phosphorescence probes and achieves a longer phosphorescence lifetime, we mixed (100 µm) complex 2 in PBS buffer, albumin solution (0.2 mg mL^−1^), and FBS (10%, *v/v*, solvent: saline). Under normoxia and 0% O_2_, we measured the phosphorescence decay traces and fit the corresponding lifetimes.

Furthermore, the phosphorescence lifetime of complex **2** in Matrigel was also studied. 0.25 mL Matrigel containing 10 ng mL^−1^ LPS, 20 ng mL^−1^ IFN‐*γ*, and 30% L929 cell‐conditioned medium was injected into the abdomen of C57BL/6‐C2J mouse. Seven days later, the mouse was euthanized with CO_2,_ and the Matrigel was taken out. The Matrigel was filled with biosynthesized collagen fibers. Then the Matrigel was incubated with complex **2** solution (100 µm) and observed with PLIM system under 20% Oxygen ambient condition and depleted oxygen with a nitrogen purge. As a control, the PLIM of pure Matrigel was also characterized.

4) In vivo Studies: The animal study was approved by the Institutional Animal Care and Use Committee (IACUC) of National Taiwan University (the assigned approval number of the laboratory: NTU107‐EL‐00135). The C57BL/6‐c2J (c2J) mice were provided by the National Taiwan University College of Medicine. About 10^5^ melanoma cells (cell lines: B16F1 mCherry) were injected subcutaneously in 4‐week‐old mice's ears. Tumor environments were imaged by two‐photon microscopy and PLIM as it grew to 1 mm in diameter. A 2% of isoflurane mixed with 98% O_2_ was used to initiate anesthesia. During the experiment, the anesthesia was achieved by injecting Avertin through IP with the proper dosage (0.25 mg g^−1^).

For the preparation of the stock solution, complex **2** was dissolved in sterilized normal saline and then pass through a syringe filter with 0.22 µm pore size. A 0.2‐mL solution was injected through the tail vein of mice. The final concentration of **2** in mice was estimated to be 100 µm. The ear was mounted on cover glasses and then observed by the multiphoton microscope. To provide two extreme oxygen levels in mice ear, on the one hand, we supplied pure oxygen gas via inhalation; on the other, we blocked the vessels at the root of the ear. Ceasing the blood circulation was done by sandwiching a bent 26G needle, and the mice ear between a coverslip and a cover glass. The needle was put upon and cross over the major vessel of the mice's ear. The additional force was applied on both sides of the glass to ensure the blockage. The color of tissue and blood was turned into white and purple, respectively, during this procedure. For the reperfusion studies, we simply increased the space between two glasses by inserting a tweezer and removed the needle simultaneously. A high tissue oxygen level was reached by the inhalation of pure oxygen (760 mm Hg). Gas was warmed to 33 °C and then delivered via a tube, which connected a mask at the end. The mask covered the entire snout of mice during the experiment. To prevent irreversible damages to the tissues, we only held the ischemic condition for 10 min. For depth‐dependent examinations, the focal plane of images was shifted 5 µm per step by a motorized objective stage. The environment was kept around 33 °C to maintain the body temperature of mice. Their vital sign was monitored during the experiment.

### Multiphoton Luminescence and PLIM Imaging System

Multiphoton luminescence imaging was performed on a commercial laser‐scanning microscope (Observer.Z1 and LSM 710, Zeiss) equipped with a micro‐incubator and a fluorescence/phosphorescence lifetime imaging system (SPC‐150, Becker & Hickl). An 80‐MHz Ti: Sapphire laser (Mai Tai DeepSee, Spectra‐Physics) was used as the excitation source. The 800 nm excitation beam was focused on samples by a 40× water immersion objective (C‐APO, Zeiss), and the average power was kept below 10 mW for in vitro study and 20 mW for in vivo study. To measure the phosphorescence decay, we turned the laser excitation on for 5 µs at a 20‐kHz gating rate by the DGG‐200 module (Becker & Hickl). This resulted in a 50 µs pixel dwell time for PLIM, which is much longer than the phosphorescence lifetime of complex **2**. During the PLIM imaging, the frame time was 3.3 s, and the overall image acquisition time was 5 min in each area to collect high enough photon counts (>100 photons) for correct curve fitting. Data analysis was performed by the software SPCImage 6.0 (Becker & Hickl).

### Retention of Complex **2** In Vivo

1.5 mM of complex 2 (solution: saline) was injected intravenously into C57BL/6‐C2J mouse via tail vein injection. Then the ear of the mouse was mounted with cover glass and observed with Nikon Inverted Multiphoton Microscope Eclipse (Nikon instrument Inc) equipped with a fluorescence/phosphorescence lifetime imaging system (Becker & Hickl) under a 40× NA = 1.15 water‐immersion objective at different time points (0, 5, 10, 15, 20, 25, 30 min, and 6 h). During the experiment, the anesthesia was achieved by injecting Avertin through IP with a proper dosage (0.25 mg g^−1^).

## Conflict of Interest

The authors declare no conflict of interest.

## Supporting information

Supporting Information

## Data Availability

Research data are not shared.
