# Supplementary material for: Functionalizing Collagen with Vessel‐Penetrating Two‐Photon Phosphorescence Probes: A New In Vivo Strategy to Map Oxygen Concentration in Tumor Microenvironment and Tissue Ischemia
Source: Adv Sci (Weinh). 2021 Aug 19;8(20):2102788. doi: 10.1002/advs.202102788 (PMC8529487; doi:10.1002/advs.202102788)
Supplement: Supplementary file 1 — Supporting Information [file ADVS-8-2102788-s001.pdf]

© 2021 Wiley-VCH GmbH

ADVANCED SCIENCE
Open Access

Supporting Information

for *Adv. Sci.*, DOI: 10.1002/advs.202102788

Functionalizing collagen with vessel-penetrating two-photon phosphorescence probes: A new *in vivo* strategy to map oxygen concentration in tumor microenvironment and tissue ischemia

Cheng-Ham Wu,[†,ξ] *Kristina S. Kisel,*[‡,§,ξ] *Muthu Kumar Thangavel,*[‡] *Yi-Ting Chen,*[†] *Kai-Hsin Chang,*[†] *Ming-Rung Tsai,*[†] *Chia-Yu Chu,*[⊥] *Yu-Fang Shen,*[♦,▽] *Pei-Chun Wu,*[#] *Zhiming Zhang,*[#] *Tzu-Ming Liu,*[*#] *Janne Jänis,*[‡] *Elena V. Grachova,*[§] *Julia R. Shakirova,*[§] *Sergey P. Tunik,*[*,§] *Igor O. Koshevoy,*[*,‡] *Pi-Tai Chou*[*,†]

Supporting Information for

Functionalizing collagen with vessel-penetrating two-photon phosphorescence probes: A new *in vivo* strategy to map oxygen concentration in tumor microenvironment and tissue ischemia

Cheng-Ham Wu, [†,ξ] *Kristina S. Kisel,* [‡,§,ξ] *Muthu Kumar Thangavel,* [‡] *Yi-Ting Chen,* [†] *Kai-Hsin Chang,* [†] *Ming-Rung Tsai,* [†] *Chia-Yu Chu,* [⊥] *Yu-Fang Shen,* [◆, ▽] *Pei-Chun Wu,* [#] *Zhiming Zhang,* [#] *Tzu-Ming Liu,** [#] *Janne Jänis,* [‡] *Elena V. Grachova,* [§] *Julia R. Shakirova,* [§] *Sergey P. Tunik,** [§] *Igor O. Koshevoy,* [*,‡] *Pi-Tai Chou** [, †]

[†] Department of Chemistry, National Taiwan University, Taipei, Taiwan

[‡] Department of Chemistry, University of Eastern Finland, 80101, Joensuu, Finland

[#] Institute of Translational Medicine, Faculty of Health Sciences, University of Macau, Macao SAR, China

[§] St.-Petersburg State University, 7/9 Universitetskaya nab., 199034, St.-Petersburg, Russia

[⊥] Department of Dermatology, National Taiwan University Hospital and National Taiwan University College of Medicine, Taipei, Taiwan

[◆] Department of Bioinformatics and Medical Engineering, Asia University, Taichung City, Taiwan

[▽] 3D Printing Medical Research Institute, Asia University, Taichung City, Taiwan

[ξ] Equal contribution

E-mail: chop@ntu.edu.tw; tmliu@um.edu.mo; stunik@inbox.ru

Figure S1. ESI-MS of complexes **1**–**3** (MeOH/H_2O).

Figure S2. The emission spectra of **1-3** in the solid-state at 298 K.

Figure S3. Effects of temperature and pH conditions on the phosphorescence decay.

Figure S4. Cell viability tests.

Figure S5. Results of *in vitro* cell studies.

Figure S6. Targetting stabilization of **2** with collagens in Matrigel.

Figure S7. *In vivo* second harmonic generation (SHG) image of collagen, two-photon phosphorescence image of the complex **2**, and combined image.

Figure S8. The retention performance of complex **2** *in vivo*.

Figure S9. *Ex vivo* calibration of complex **2** in collagen fiber under various oxygen concentrations.

Figure S10. *In vivo* images of various Re complexes.

Table S1. Phosphorescence lifetime of **2** in various biological substances.

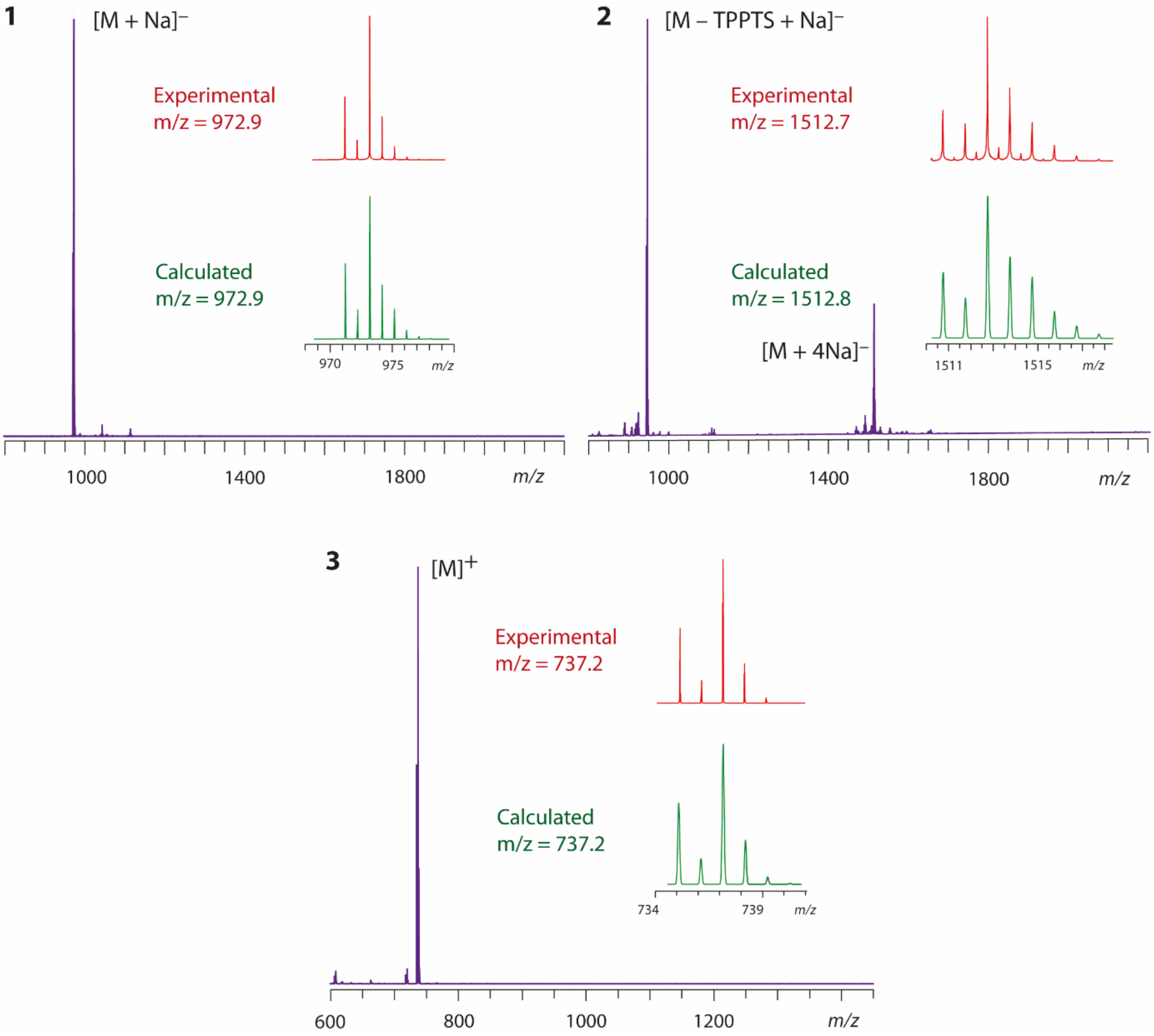

Figure S1. ESI-MS of complexes **1–3** (MeOH/H_2O).

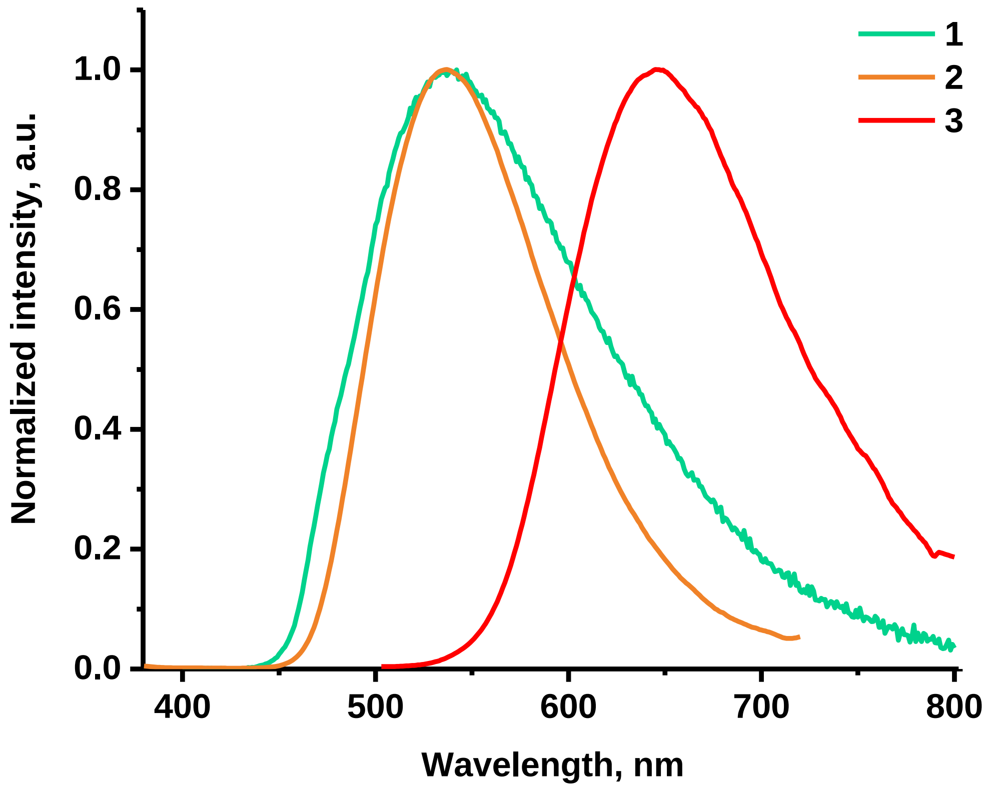

Figure S2. The emission spectra of **1-3** in the solid-state at 298 K. λ_{ex} = 375 nm

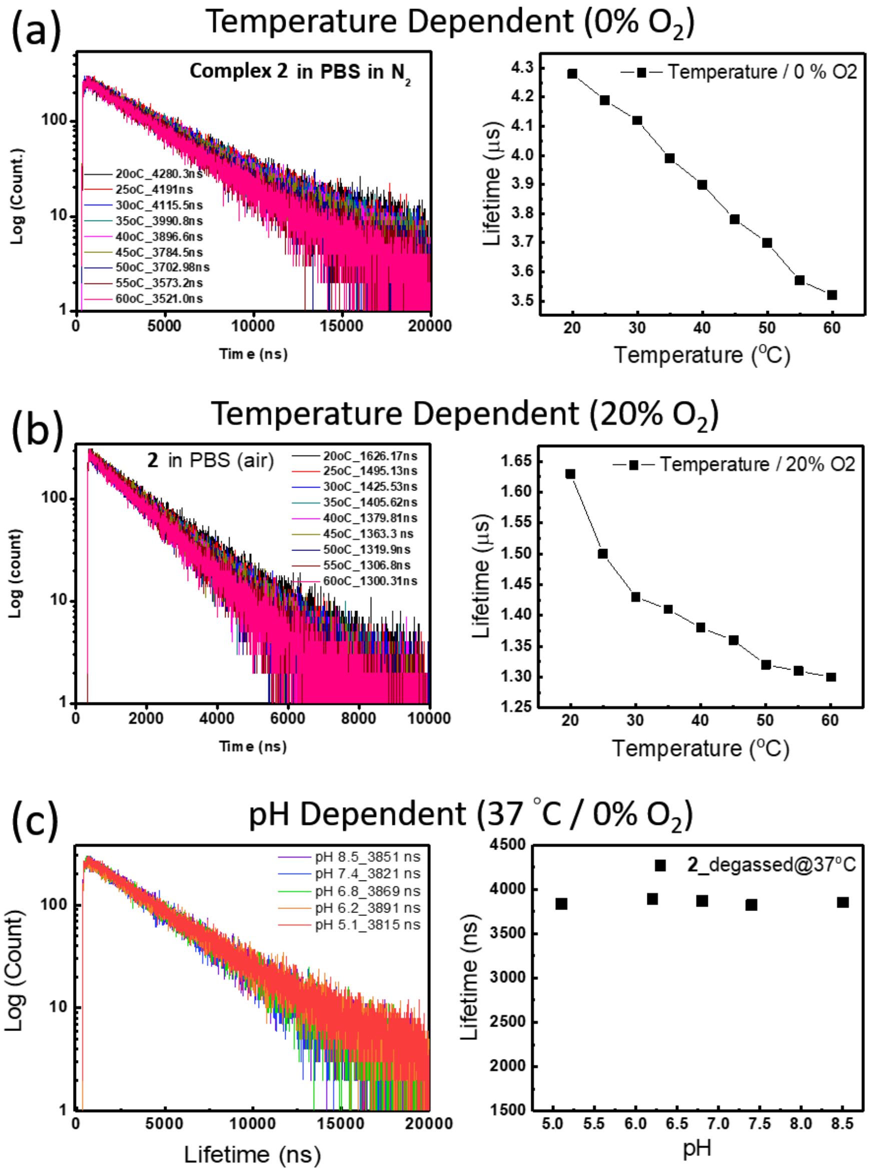

Figure S3. Effects of temperature and pH conditions on the phosphorescence decay. The temperature-dependent phosphorescence lifetime of complex **2** dissolved in water purged with (a) nitrogen (0% Oxygen) and (b) 20% Oxygen gas. (c) The phosphorescence lifetime of complex **2** in water with pH = 5.1, 6.2, 6.8, 7.4, and 8.5.

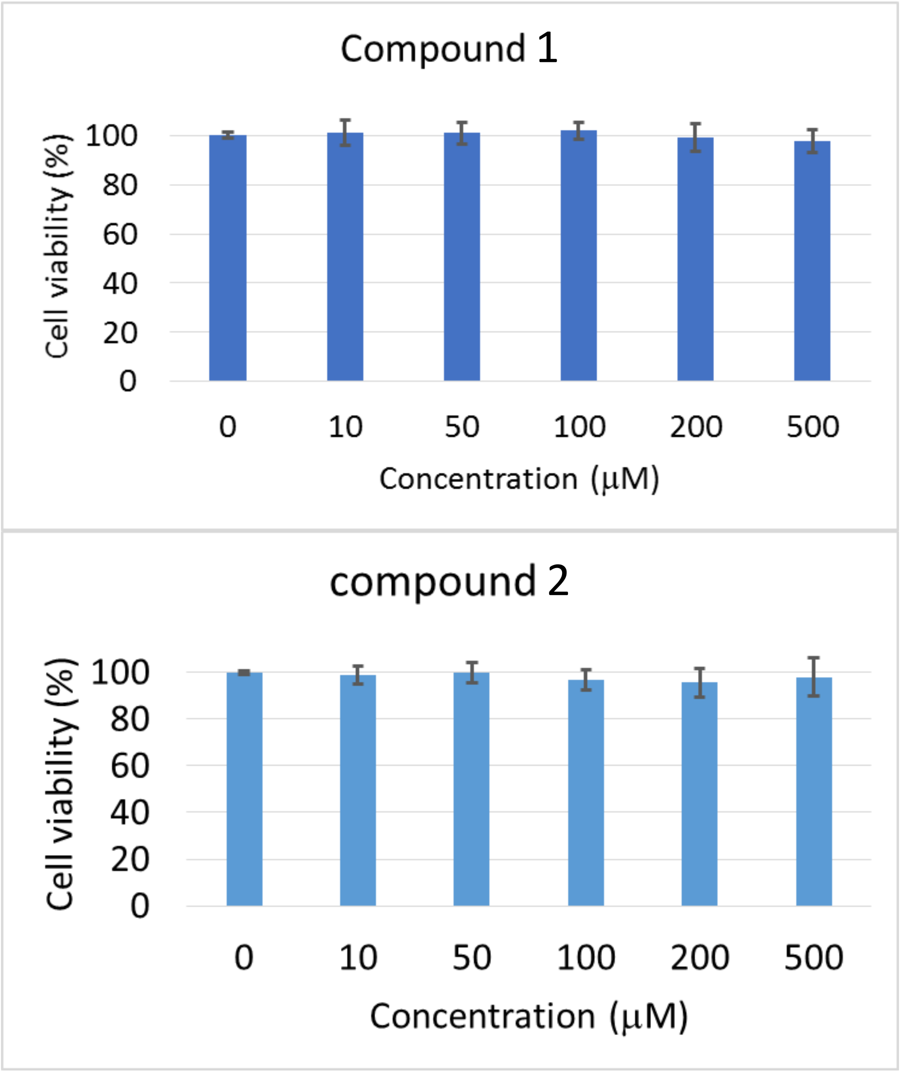

Figure S4. Cell viability tests. MTT assay for the viability of HepG2 cells treated with various concentrations of complexes **1** and **2**. Error bars represent the standard deviations of three measurements.

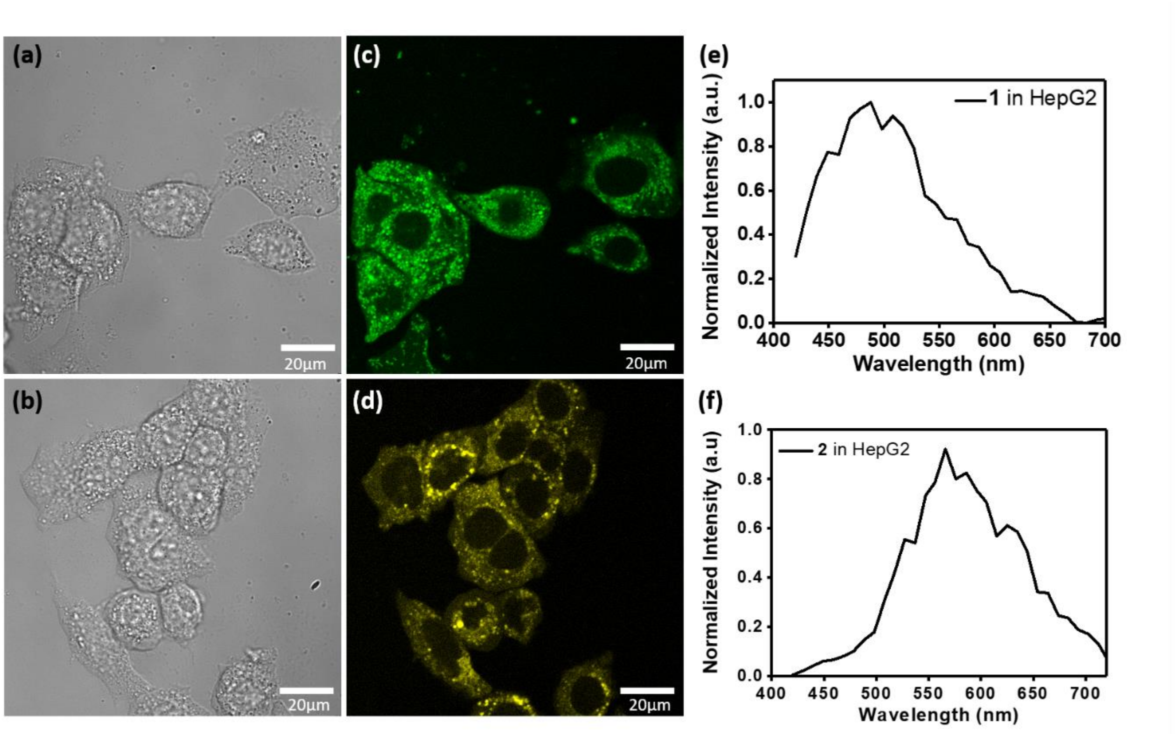

Figure S5. Results of *in vitro* cell studies. The (a, b) bright-field images, (c, d) two-photon luminescence images, and (e, f) the two-photon luminescence spectra of HepG2 cells incubated for 15 h with 150 μM compound **1** (upper row) and **2** (lower row) at 37 ^{o}C, 5% CO_2 condition.

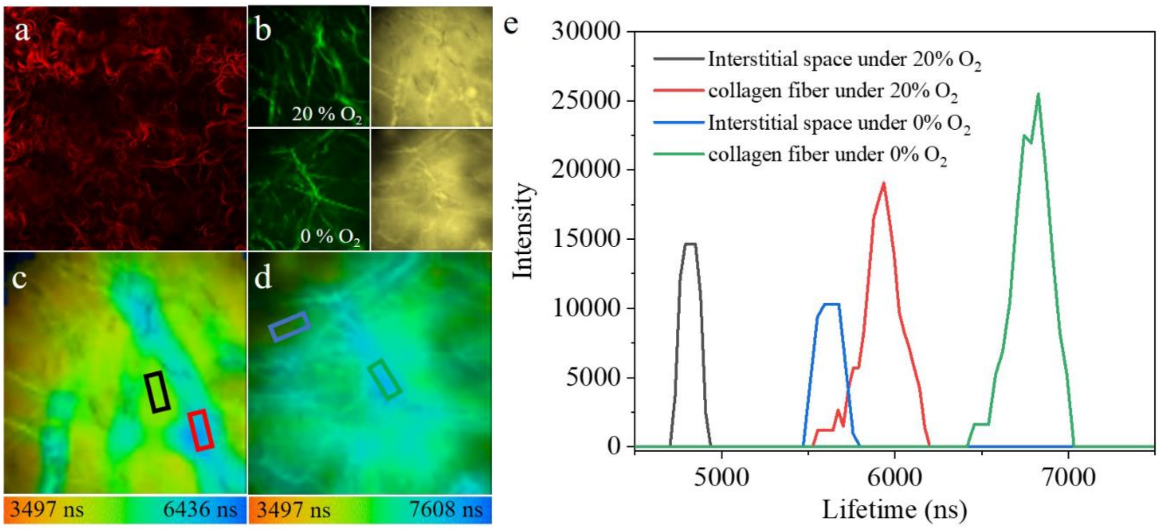

Figure S6. (a) Second harmonic generation imaging (SHG) of collagen networks in a Matrigel, plugged in a mouse's abdomen cavity for 7 days. Fields of view: 265 × 265 μm (b) The SHG image of collagens (green color) and the two-photon phosphorescence image of the complex **2** in the Matrigel under 20% O_2 and 0 % O_2 conditions. Fields of view: 85 × 85 μm. (c)-(d) The two-photon phosphorescence lifetime imaging microscopy of (b). (e) the lifetime histograms

in the regions with collagen fibers (red and green square) and interstitial spaces (black and blue square).

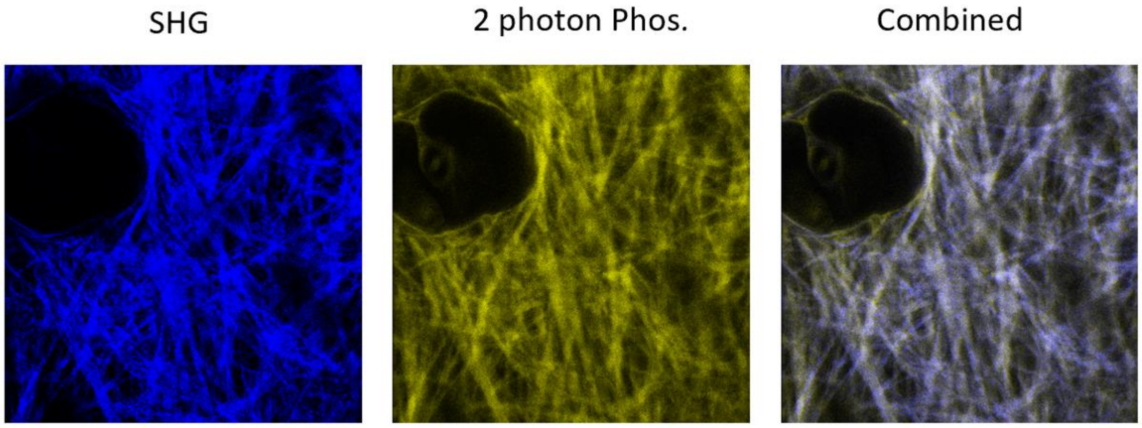

Figure S7. *In vivo* second harmonic generation (SHG) image of collagen (blue color), two-photon phosphorescence image of the complex **2** (yellow color), and combined image. The excitation wavelength is 800 nm. Fields of view: 100 × 100 µm.

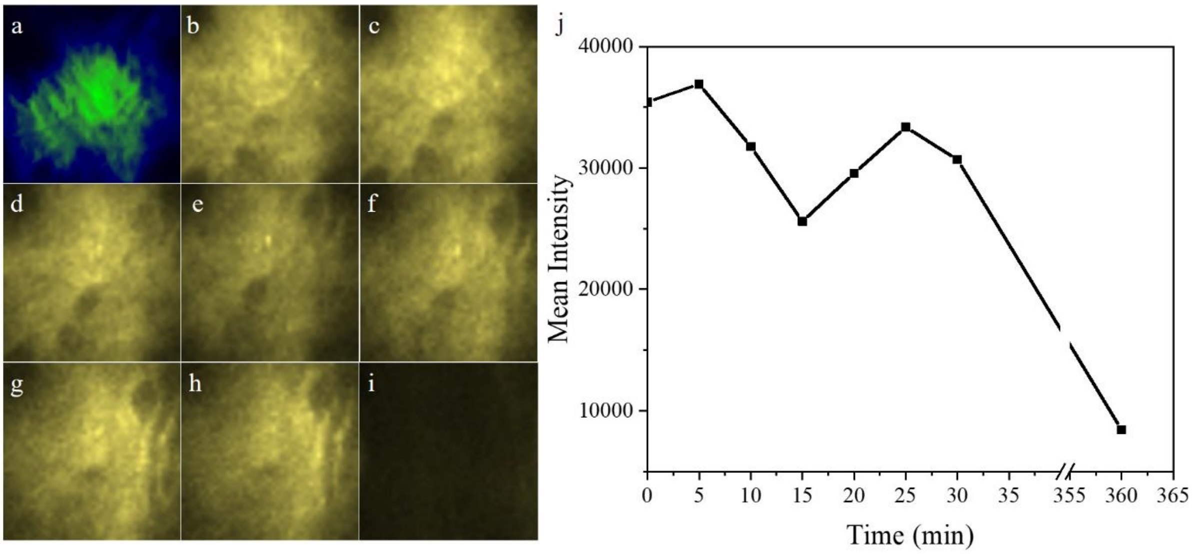

Figure S8. (a) The 800 nm excited second harmonic generation (green) and two-photon fluorescence imaging (blue). (b-i) The time-course two-photon phosphorescence intensity images of complex **2** *in vivo*. (j) The average intensity of (b-i) over time.

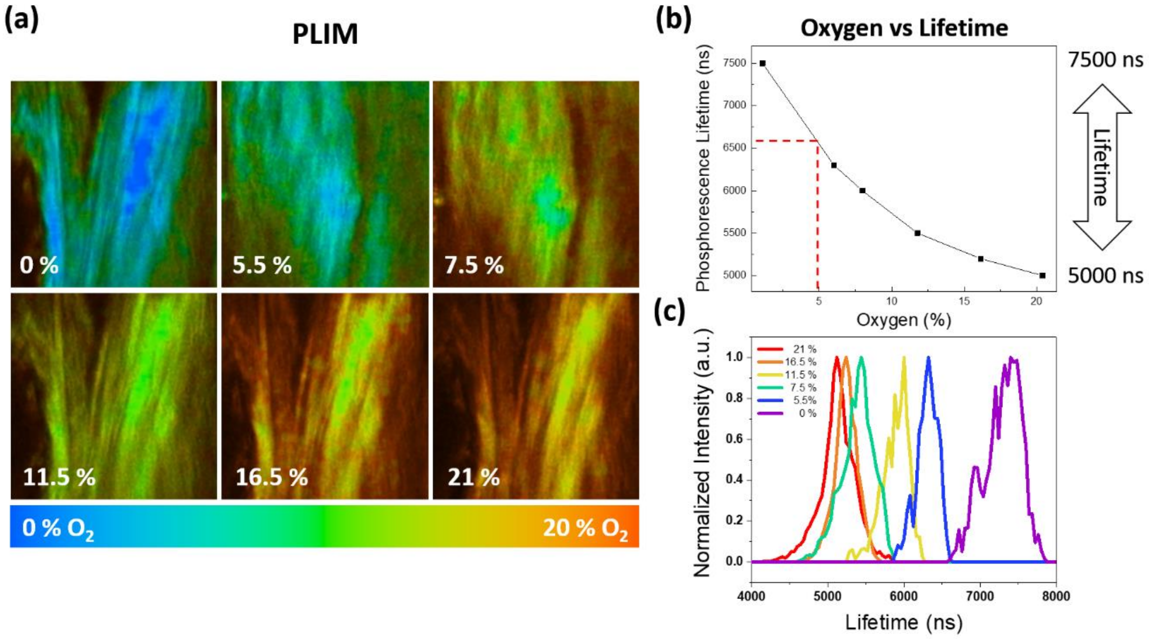

Figure S9. *Ex vivo* calibration of complex **2** in collagen fiber under various oxygen mole fraction. (a) The PLIM images of complex **2** in *ex vivo* tissues at 0%, 5.5%, 7.5%, 11.5%, 16.5%, and 21% oxygen mole fraction. (b) The plot of phosphorescence lifetime versus the oxygen mole fraction. Lifetimes increase from 5 to 7.5 μs as the oxygen mole fraction decreases. (c) Lifetime histograms in collagen under various oxygen mole fraction. Fields of view: 100 × 100 µm.

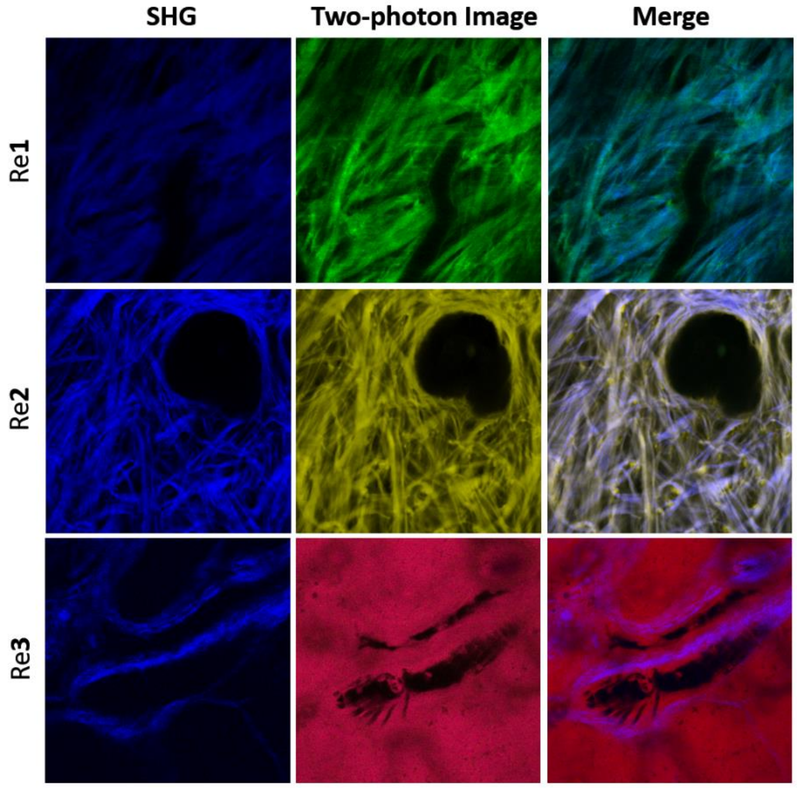

Figure S10. *In vivo* images of various Re complexes under excitation with λ_{exc} = 750 nm for **1,** and λ_{exc} = 800 nm for **2** and **3**. Second-harmonic generation (blue color) and two-photon phosphorescence images of Re complexes **1**(green color), **2**(yellow color), and **3**(red color). The TPPTS ligand on **1** and **2** colocalizes well with collagen, while the PTA one (**3**) does not and tends to spread all over the extravascular space. Fields of view: 240 × 240 µm.

Table S1. Two-photon phosphorescence lifetime of **2** in various biological substances

	Lifetime (ns)	
Sample	20% O_2	0% O_2
PBS buffer	1565	3788
Albumin solution	1608	3487
FBS solution	1841	3767
Pure Matrigel	1627	2278
